# Cell-based therapy in lung regenerative medicine

**DOI:** 10.1186/2050-490X-2-7

**Published:** 2014-04-11

**Authors:** Jibing Yang, Zhenquan Jia

**Affiliations:** Division of Pulmonary and Critical Care Medicine, Department of Internal Medicine, University of Michigan, Ann Arbor, MI 48109 USA; Department of Biology, College of Arts & Sciences, University of North Carolina at Greensboro, Greensboro, NC 27412 USA

**Keywords:** Lung regenerative medicine, Stem cells, Lung injury, Lung epithelial stem/progenitor cells, Mesenchymal stem cells, Endothelial progenitor cells, Therapy

## Abstract

Chronic lung diseases are becoming a leading cause of death worldwide. There are few effective treatments for those patients and less choices to prevent the exacerbation or even reverse the progress of the diseases. Over the past decade, cell-based therapies using stem cells to regenerate lung tissue have experienced a rapid growth in a variety of animal models for distinct lung diseases. This novel approach offers great promise for the treatment of several devastating and incurable lung diseases, including emphysema, idiopathic pulmonary fibrosis, pulmonary hypertension, and the acute respiratory distress syndrome. In this review, we provide a concise summary of the current knowledge on the attributes of endogenous lung epithelial stem/progenitor cells (EpiSPCs), mesenchymal stem cells (MSCs) and endothelial progenitor cells (EPCs) in both animal models and translational studies. We also describe the promise and challenges of tissue bioengineering in lung regenerative medicine. The therapeutic potential of MSCs is further discussed in IPF and chronic obstructive pulmonary diseases (COPD).

## Review

### Introduction

The lung disease has become one of the major public health issues across the world with the increased human activities, environmental changes, air pollution, smoking, and various pathogens like influenza. The World Health Organization ranks lung diseases second in epidemiology, mortality and cost and predicts that about one fifth deaths will be attributed to lung diseases by 2020 [1]. Currently, there are no therapeutic ways to inhibit or reverse the pathobiology of many destructive lung diseases. These include chronic obstructive pulmonary disease (COPD) which is the leading cause of death in pulmonary diseases worldwide, idiopathic pulmonary fibrosis (IPF), cystic fibrosis, pulmonary hypertension (PH) and the acute respiratory distress syndrome (ARDS) [2]. Lung transplantation becomes the only choice for many patients. However, many patients die in the waiting period due to the shortage of available donor lungs. Furthermore, the average of survival time post-transplantation for recipients is around 5–6 years [3]. Long-term graft dysfunction and bronchiolitis obliterans syndrome (BOS) are still the major obstacles to be overcome post transplantation [4]. Thus, new and innovative options are in urgent need for those patients.

Recent progresses in stem cell research allow investigators to study cell-based therapies in the treatment of lung diseases. Stem cells are a population of undifferentiated cells characterized by three main functions: 1) ability to divide asymmetrically (called self-renew); 2) clonality generally arising from a single cell; and 3) potency to differentiate into different type of cells or tissues [5]. Pluripotent stem cells have the ability to generate all lineages of body and include embryonic stem (ES) cells and induced pluripotent stem (iPS) cells [6]. ES cells are first derived from the inner cell mass at the blastocyst-stage embryo. A good example of iPS is the Yamanaka experiment in which mouse and human fibroblasts were transfected with four transcription factors (OCT3/4, SOX2, c-MYC, KLF4) to reprogram the somatic cells into iPS [7]. Lung stem cell research slightly lags behind studies on other organs. There is relatively limited knowledge about the endogenous progenitor cells of human lung tissue until recently. Moodley and colleagues performed a xenograft implantation in which they injected human amnion epithelial cells parenterally into bleomycin-treated severe combined immunodeficiency (SCID) mice as pulmonary fibrosis model and demonstrated reduced inflammation and fibrosis [8]. The injected cells also developed an alveolar epithelial phenotype with lamellar body formation and expression of surfactants A and D. More interesting, Kajstura and coworkers recently identified stem cells from adult human lung tissue using the stem cell antigen marker c-kit [9]. After being administrated into an injured mouse lung, the cells demonstrated pluripotent capability of generating human bronchioles, alveoli, and pulmonary vessels within the damaged organ. Although some disputes exist on tissue-specific adult human lung stem cells in Kajstura study [10, 11], these evidences collectively suggest that isolated adult lung stem cells are potentially of great clinical importance for cell-based therapies in pulmonary diseases.

This review will briefly highlight the recent progresses on mouse lung stem cell research and its applications in some clinical trials for specific lung diseases such as IPF and COPD. We will mainly discuss the endogenous lung stem cells named as epithelial stem/progenitor cells (EpiSPCs) in different anatomic locations, mesenchymal stem cells (MSCs), and endothelial progenitor cells (EPCs). In addition, we will briefly discuss novel bioengineering approaches to generate implantable lung tissues or organs *ex vivo* and *in vivo*. Stem cells from different origins may be repopulated into the decellularized or bioengineered scaffold to create new functional organs.

### Lung stem cells: classification, origin, biomarkers, and function

Recent studies mainly in mice have identified several adult stem cell lines in distinct anatomic locations of lung [12–14]. There might be some equivocal terms to define each cell line. In general, these diverse cell lines can be called lung endogenous stem/progenitor cells. According to the ability to differentiate, all stem cells can be categorized into 5 groups: totipotent, pluripotent, multipotent, oligopotent and unipotent [15]. Most lung endogenous stem/progenitor cells belong to multipotent or oligopotent cells. Alveolar epithelial cells (AECs) type II are unipotent since they only differentiate into type I cells [16]. Here we will mainly focus on three extensively studied populations of lung adult stem/progenitor cells in terms of their origin, biomarkers, and function mainly restricted to animal models.

### Epithelial stem/progenitor cells (EpiSPCs)

Region-specific endogenous EpiSPCs in the adult lung has been extensively reviewed recently [2, 14, 17, 18]. Briefly, different lineages of EpiSPCs reside in the proximal trachea, bronchi, bronchioles, and alveoli regions to maintain local epithelial homeostasis and repair. Basal cells in tracheobronchial region express transcription factors Trp63, cytokeratin Krt5, and surface receptor Ngfr [19]. Two main types of secretary cells along the proximal-distal axis include the secretoglobin family 1A member 1-positive clara cells (Scgb1a1^pos^, also known as CCSP or CC10) and mucus/goblet cells Muc5AC^pos^ and Muc5B^pos^[20]. Bronchioalveolar stem cells (BASCs) at the bronchioalveolar duct junction of terminal bronchioles, also termed double-positive cells, express CCSP and surfactant protein C [21]. More recently, two independent groups utilized SFTPC-CreER system to track AEC type II and demonstrated that type II cells give rise to AEC type I [22, 23]. The α6β4 positive AECs express little or none of CC10 or pro-SPC. Barkauauskas and colleagues confirmed that SPC^pos^ AECs type II self-renew and maintain differential potential over one year [16]. PDGFRα^pos^ lung stromal cells or lopofibroblasts may contribute to a stem cell niche of Type II cells to facilitate their growth and differentiation, suggesting that direct contact between AECs and mesenchymal cells or a paracrine effect is necessary to activate or initiate cell proliferation and differentiation. One study by McQualter and colleagues supports the idea in which they isolated EpiSPC expressing EpCAM, CD24, CD45, CD31, and Sca-1 from enzymatically digested adult mouse lung tissue [24, 25]. Either co-culture this population with EpCAM^neg^ Sca-1^Pos^ lung mesenchymal cells or directly adding soluble fibroblast growth factor (FGF)-10 and hepatocyte growth factor (HGF) promoted clonal proliferation and self-renewal.

Although there are many endogenous epithelial progenitor cell lineages in the adult lung, bone-marrow derived progenitor cells represent a distinct origin and have been shown to migrate to the airway epithelium and contribute to tissue repair in lung disease models. Wong and colleagues identified a bone-marrow-derived progenitor cells in mice and human that expressed CCSP and hematopoietic marker CD45 and mesenchymal origin (CD73, CD90, CD105) [26]. CSSP^+^ cells expressed basal cell markers and surfactant protein A-D when cultured at the air-liquid interface *ex vivo*. These cells homed to injured airway in response to naphthalene-induced lung damage. Moreover, in the sex-mismatched bone marrow transplantation, Y chromosomes were found to be widespread in type II cells in female recipient lung [27]. Taken together, these observations strongly suggest that except lung endogenous epithelial progenitor cells participating in repair, bone marrow-derived progenitor cells may also home to sites of injury and differentiate into epithelial phenotype.

### Mesenchymal stem cells (MSCs)

MSCs was first isolated from bone marrow and described by Friedenst in 1968 as a plastic adherent fibrablast-like appearance and distinct from haematopoietic stem cells (HSCs) [28]. Thereafter, MSCs has been isolated from many other tissues, including umbilical cord blood, placenta, adipose tissue, amniotic fluid, Wharton’s jelly [29, 30]. MSCs are multipotent and can differentiate into cells of various tissues like bone, cartilage, muscle, liver, and lung [13]. There is no consistent cell surface markers to isolate MSCs from different tissues, though the Mesenchymal and Tissue Stem Cell International Committee has proposed minimal criteria to define human MSCs [31]. Lineage tracing studies with gene fibroblast growth factor (FGF)-10 have disclosed that at least two distinct populations of MSCs residing at the trachea and branching tip of the epithelium along the airway differentiate into smooth muscle cells [32]. Further studies demonstrated that MSCs can be enriched with cell surface markers CD45^neg^ CD31^neg^ EpCAM^neg^ Sca-1^pos^ and play an important role as the epithelial progenitor cell niche to support their proliferation and differentiation *in vitro*[24, 33]. More interesting, Chow et al. defines the lung MSCs in the conditional knock-out mice EC-SOD^-/-^ under the control of ABCG2 promoter as multipotent vascular precursors and finds that lung MSCs differentiate into myofibroblast, endothelial and pericytes *in vitro*[34]. Lama and colleagues successfully isolated MSCs from the bronchoalveolar lavage of adult human lung allografts [35]. These MSCs express common messenchymal markers CD73, CD90, and CD105, but are absent of hematopoietic lineage markers CD14, CD34, and CD45. They are capable of differentiating into adipocytes, chondrocytes, and osteocytes. Although adult lung contains some lineages of MSCs, more studies focus on the BM-derived MSCs in human disease models. Gazdhar and colleagues recently isolated a population of human hepatocyte growth factor (HGF)-expressing stem cells at fibrotic area in usual interstitial pneumonia patients. These HGF-positive cells co-expressed MSC markers CD44, CD29, CD105, CD90 and CXCR4 indicating bone-marrow (BM) derived stem cell characteristic [36]. In order to understand whether they have anti-fibrotic property, they instilled BM-MSCs transfected with HGF into rat lung 7 days after bleomycin treatment and observed an attenuated fibrosis. Although the exact mechanisms of how MSCs contribute to beneficial effects against diseases are not yet fully understood, a growing body of evidences supports a paracrine effect to repair injured cells. Human MSCs condition media facilitates wound repair in human type II cell lines A549 and primary human small airway epithelial cells *in vitro* by producing secretome containing proteins such as fibronectin, lumican, periostin, and IGFBP7 [37]. Similar studies in cardiac fibrosis shows MSCs condition media inhibited cardiac fibroblast proliferation by up-regulated cell-cycle arresting genes such as elastin, myocardin, DNA-damage inducible transcripts [38, 39]. The above properties make MSCs an attractive therapeutical reagent to treat many chronic diseases in clinical trials as discussed later in this article.

### Endothelial progenitor cells (EPCs)

Study on EPCs was first reported by Asahara and colleagues in 1997 by the discovery of HSCs capable of differentiation into an endothelial phenotype [40]. EPCs can be recruited from bone marrow to injured lung tissue. Although the mechanisms are not fully understood, the released cytokines hypoxia inducible factor (HIF) and vascular endothelial growth factor (VEGF) may play a role in the recruitment of EPCs to sites of hypoxia from bone marrow [41]. When EPCs migrated into hypoxic destinations, they have the capacity to differentiate into endothelial cells and generate new blood vessels [42]. The phenotypic identification of EPCs is currently inconsistent because there is a lack of a unique combination of marker proteins to define EPCs. According to the Duda protocol, EPCs can be isolated by FACS through a population of CD31^+^/CD34^bright^/CD45^dim^/CD133^+^[43]. A general combination of shared markers for EPCs includes stem cell marker CD133 (also known as AC133), endothelial cell marker VEGFR2, as well as hematopoietic marker CD34 [44]. Ribattihas et al. proposed a way to divide EPCs into two different subpopulations, early (CD34^+^ CD31^+^ CD14^+^) and late (CD31^+^ CD144^+^ CD146^+^ CD105^+^ CD45^-^ CD14^-^ CD115^-^) EPCs, with distinct cellular morphology, growth pattern and abilities to secrete angiogenic factors [45, 46]. Currently, it is still unable to discriminate BM or peripheral blood derived EPCs from lung resident EPCs. This may prevent us from further understanding the contribution of endogenous EPCs in lung vascular disease repair and regeneration.

Large numbers of animal and clinical studies have been performed to investigate the beneficial roles of EPCs in lung diseases. Animal studies have shown that BM-derived EPCs can home to site of ischemia, and newly formed vessels were detected at ischemic locations [47]. For example, in animal models of monocrotaline (MCT)-induced pulmonary hypertension (PH), transplantation of BM-derived EPCs prevented PH and restored microvascular architecture and perfusion in rats and dogs [48, 49]. However delayed administration of EPCs at 3 weeks post MCT treatment partially prevented the increase in right ventricular systolic pressure compared to a shorter time point [48]. When the endothelial nitric oxide synthase (*eNOS*) gene was transfected into EPCs as a vehicle, they found that *eNOS* gene therapy significantly reversed an established disease in this model. Acute lung injury (ALI) and end stage ARDS are among the most common causes of death in ICU. During the acute exudative stage, endothelial cells can be detached from the pulmonary vessels and thus appear in the peripheral blood circulation. EPCs transplantation represents an innovative treatment option for the *de novo* formation of blood vessels. Many clinical studies focused on quantification of peripheral EPCs and their correlations with disease outcomes. However, Suratt and colleagues first studied the capacity of EPCs to replenish lung cells indirectly by administration of allogeneic HSCs [50]. They found that lung biopsies from female recipients contain male donor-derived epithelial and endothelial chimerism. Except these promising preclinical and clinical studies, several early phase clinical trials on EPCs transplantation were underway. One pilot trial by Wang and colleagues showed that transplantation of autologous EPCs into patients with idiopathic pulmonary arterial hypertension significantly improved 6-minute walk capacity and hemodynamic function without obvious adverse effects [51]. More recently, a phase I clinical trial (ClinicalTrials.gov Identifier: NCT00469027) of transplantation of autologous EPCs transfected with *eNOS* gene to patients with severe pulmonary arterial hypertension was completed. Safety concern was not noticed during the trial and patients appeared well tolerated with the genetically engineered EPC [2]. The complete results of the trial will be released soon. Although recent studies indicate that both circulating and lung residential EPCs facilitate tissue repair and regeneration, more knowledge on optimal cell preparation, storage, dosage, administration route and time is strongly needed for future clinical application. Finally, a comprehensive assessment of EPCs can be referred to other reviews [13, 44, 52].

### Tissue bioengineering

Tissue bioengineering is defined as the generation of functional tissue for replacement of injured or pathological tissue. Adult lung is architecturally complex as a hierarchical model of homoeostasis, and is made up of more than 40 distinct types of cells [53]. It is impractical to directly build a functional whole lung organ with current knowledge and technology. However, it is still feasible to produce part of the upper, lower airway or the alveolar tissue. In fact, significant progress has recently been achieved using decellularized or synthetic scaffolds to generate tracheal cartilage as well as tendon tissue in diaphragm for clinical application [54, 55]. Epithelial cells and MSC-derived chondrocytes were implanted to the decellularized donor trachea and restored the trachea function in the recipient [56]. The generation of lower airway and alveolar tissues is more challenging and restricted to animal studies presently. Seeding somatic lung progenitor cells onto synthetic polymer scaffold *in vitro* or implanted *in vivo* promoted cell differentiation [57]. However, the *in vivo* transplantation caused an inflammatory response which disrupted lung development. A recent pioneer work by Peterson and colleagues demonstrated that a bioengineered lung achieved gas exchange upon reimplantation in rats *in vivo*[58]. They first decellularized the rat lung with detergents to remove all immunogenic cells. Neonatal epithelial and vascular endothelial cells were seeded into the corresponding anatomic locations in the scaffold cultured in a bioreactor which contained suitable cell growth media. After cultured for several days, the engineered lungs were transplanted into syngeneic rat for 45–120 minutes and recorded gas exchange. Taken together, these preclinical studies indicate a bright future for bioengineered lung tissues in regenerative medicine. However, a number of key challenges still need to be resolved before initiating any clinical trial. These mainly include the optimal origin of progenitor stem cells (EpiSPCs, MSCs, EPCs, and/or other origins), extracellular matrix components, potential immunogenicity, ideal *in vitro* culture condition, and other implantation-related dosage, order, route, function monitoring system [59, 60].

### Lung diseases with cell-based therapies

Idiopathic pulmonary fibrosis (IPF) is a fatal form of pulmonary fibrosis disease characterized by extracellular matrix deposition and scar tissue formation in the interstitial lungs over time. The incidence of IPF is 13 to 20 cases per 100,000 people [61]. Typically the disease is found in old adults and the median survival time is 3–5 years. The clinical presentation includes exertional dyspnea, cough, functional and exercise limitation, impaired quality of life and risk for acute respiratory failure and death. Unfortunately, there is no FDA approved treatment or cure for IPF patients. Pirfenidone is an approved anti-fibrotic and anti-inflammatory drug in Europe and Japan, but still in clinical trials in North America [62, 63]. Although the etiology and pathogenesis of IPF is not fully understood, the accumulation of activated myofibroblasts is thought to be the source of interstitial collagens. There are at least four proposed cellular origins of myofibroblasts including expansion of lung residential fibroblasts, pericytes, recruitment of BM-derived fibrocytes, and alveolar epithelial cells undergoing epithelial-mesenchymal transition (EMT) [64, 65]. The circulating fibrocytes have been proposed as a clinical marker to assess disease progress [66].

Recently, stem cell therapy has emerged as a critical treatment for many chronic lung diseases. Murine bleomycin model is one of the best characterized animal models for human IPF disease. With this model, several groups have shown that administration of allogeneic BM-MSCs reduced inflammation and collagen deposition [67–69]. Other source of stem cells from placenta and human umbilical cord also demonstrated reduced lung tissue damage in the mouse bleomycin models [70, 71]. More interesting, HSCs and MSCs have been genetically manipulated as vehicles to deliver keratinocyte growth factor (KGF) into the lung injured sites. HSCs provided better protection from bleomycin-induced injury and promoted endogenous type II AECs proliferation while MSCs delivery just reduced collagen 1α1 mRNA [72]. These preclinical studies in animal models strongly suggest that MSCs may be effective to treat human IPF. Furthermore, allogeneic MSCs was safe and well tolerated to treat refractory lupus erythematosus in a recent clinical trial [73]. Considering the potential beneficial role of MSCs in pre-clinical models, a few clinical trials on IPF patients have been approved. One recently completed Phase 1b trial by Tzouvelekis and colleagues that was aimed to determine the safety of endobronchial administration of autologous adipose derived stromal cells-stromal vascular fraction to IPF patients with mild to moderate severity demonstrates an acceptable safety profile [74]. The treatment efficacy will be evaluated in a future trial within a large population of patients. In addition, FDA has approved another clinical trial phase I study of autologous MSCs to treat IPF patients (initiated in March 2013, ClinicalTrials.gov Identifier: NCT01919827). This pilot trial will evaluate the safety and feasibility of the endobronchial administration of autologous BM-MSCs in patients with mild to moderate IPF. This historical step illuminates the great potentiality of cell-based therapy in respiratory regenerative medicine. On the other hand, two separate groups found that MSCs may play a role in the fibrogenic process of the lung. Antoniou and colleagues reported an increased expression of the axis stromal-cell-derived factor-1 (SDF-1)/CXCR4 in BM-MSCs from IPF patients, suggesting the BM-MSCs may probably implicate in the pathogenesis of IPF by recruiting MSCs to lung injury sites [75]. Walker and colleagues recently found that allografted-derived local MSCs contain a profibrotic phenotype by up-regulation of FOXF1, α-SMA, and collagen I in human lung transplant recipients with BOS [76]. Overall, further studies are still necessary to completely understand the exact role of MSCs in the pathophysiology of IPF.

*COPD* is becoming a major devastating disease worldwide. It is characterized by progressive poor airflow caused by chronic small airway inflammation (known as chronic bronchitis) and destruction of lung tissue (known as emphysema). Cigarette smoke is the major risk factor to induce the chronic inflammation and finally destructs bronchial and alveolar epithelial cells [77]. Repairing the destroyed lung structure with stem cell therapies becomes an extremely attractive choice to treat COPD. MSC is the most extensively studied candidates in clinical trials not only for COPD, but also for other chronic diseases [78–80]. One recently completed placebo-controlled, randomized trial in 62 patients concluded that systemic administration of MSCs was safe in moderate to severe COPD patients [81]. However, they did not observe significant improvement in lung function or quality of life within the 2-year follow-up period after MSCs treatment, except reduced level of C-reactive protein. Further studies with MSCs or other populations of lung endogenous stem cells in more patients are needed to evaluate in depth the efficacy and safety of cell therapies in COPD patients.

## Conclusion

Stem cell therapy appears to be a promising strategy to attenuate or even reverse chronic lung diseases. Over the past decade, numerous preclinical studies have demonstrated the capability of EpiSPCs, MSCs, and EPCs from adult lung to facilitate tissue repair and regeneration in a number of pulmonary disease models. Many completed or ongoing clinical trials have supported their safety in the treatment of lung diseases. Another rapid growing field of lung bioengineering offers further promise in lung regenerative medicine. However, there are more unanswered questions in this field than what we currently know. For example, how to achieve stem cell stable growth and differentiation *in vitro* and *in vivo*? Can we completely ignore the immune rejection? What about the long-term possibility of tumorigenesis? Overall, stem cell therapy in lung regenerative medicine is still in its infancy and many challenges remain scientists to explore.

## Abbreviations

AECs: Alveolar epithelial cells
ARDS: Acute respiratory distress syndrome
BM: Bone marrow
BASCs: Bronchioalveolar stem cells
BOS: Bronchiolitis obliterans syndrome
*CFTR*: cystic fibrosis transmembrane conductance regulator protein
COPD: Chronic obstructive pulmonary disease
EMT: Epithelial-mesenchymal transition
eNOS: endothelial nitric oxide synthase
EPCs: Endothelial progenitor cells
EpiSPCs: Epithelial stem/progenitor cell
ES: Embryo stem cells
FGF: Fibroblast growth factor
HGF: Hepatocyte growth factor
HIF: Hypoxia inducible factor
HSCs: Haematopoietic stem cells
IPF: Idiopathic pulmonary fibrosis
iPS: induced pluripotent stem cells
MCT: Models of monocrotaline
MSCs: Mesenchymal stem cells
PH: Pulmonary hypertension
SCID: Severe combined immunodeficiency
SDF-1: Stromal-cell-derived factor-1
VEGF: Vascular endothelial growth factor.
